# Holistic Optimization toward Ultrathin Flexible Perovskite Solar Cells with High Efficiency and Mechanical Robustness

**DOI:** 10.1002/advs.202415372

**Published:** 2025-06-02

**Authors:** Guanqi Tang, Fangyuan Zheng, Jiajun Song, Qidong Tai, Jiong Zhao, Feng Yan

**Affiliations:** ^1^ Research Institute of Frontier Science Southwest Jiaotong University Chengdu 610031 P. R. China; ^2^ Department of Applied Physics Research Center for Organic Electronics The Hong Kong Polytechnic University Hung Hom Kowloon Hong Kong 999077 P. R. China; ^3^ The Institute of Technological Sciences Wuhan University Wuhan P. R. China 430072; ^4^ Research Institute of Intelligent Wearable Systems The Hong Kong Polytechnic University Hung Hom Kowloon Hong Kong SAR 999077 P. R. China

**Keywords:** lightweight, mechanical robustness, PEDOT:PSS electrode, strain release, ultrathin perovskite solar cells

## Abstract

An ultrathin and flexible perovskite solar cell (f‐PSC) is highly desirable as a portable power source, while the rigidity of key components including perovskite and transparent electrode of a device leads to challenges in fabrication. Here, several approaches are developed to improve the mechanical flexibility and photovoltaic performance of ultrathin f‐PSCs. First, a two‐dimensional perovskite with low Young's modulus is introduced at the boundaries of perovskite films as a lubricant to release stress which is confirmed by in situ TEM characterization. Second, conductive PEDOT:PSS doped with sucralose is used as a transparent electrode to enhance the mechanical flexibility and photovoltaic performance of the device. Third, an ultrathin PET substrate is employed to shift the neutral plane into the perovskite film which further improves the mechanical flexibility of devices. Consequently, an ultrathin f‐PSC is successfully fabricated with a power conversion efficiency of 21.44% and a record power‐per‐weight value of 47.8 W g^−1^. A stretchable device is realized by laminating the ultrathin f‐PSC on a pre‐strained substrate, which shows stable performance when it is stretched up to 40%. The f‐PSC shows a high efficiency of 36.25% under room light intensity, suggesting great potential for indoor photovoltaic application.

## Graphical abstract

In this manuscript, 2D perovskite grain boundaries are constructed to improve the intrinsic flexibility of perovskite films by efficiently releasing the strain. In addition, a novel additive, sucralose, is employed to enhance the conductivity and mechanical flexibility of PEDOT:PSS electrodes. Consequently, an ultrathin f‐PSC is successfully fabricated with a power conversion efficiency of 21.44% and a record power‐per‐weight value of 47.8 Wg‐1.

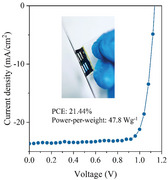

## 1 Introduction

Perovskite solar cells (PSCs) have emerged as a next‐generation photovoltaic technology due to their convenient low‐cost fabrication and high power conversion efficiencies (PCEs) exceeding 26% thus far.^[^ ^1^, ^2^, ^3^, ^4^, ^5^ ^]^ In addition, the low‐temperature processing and thin film thickness of perovskite films enable the fabrication of flexible and lightweight devices, which can harvest solar energy on non‐planar and mobile structures and serve as portable power sources for building integrated photovoltaics, near‐space vehicles, wearable electronics and internet of things (IOT).^[^ ^6^, ^7^, ^8^, ^9^, ^10^ ^]^ Flexible PSCs (f‐PSCs) are usually fabricated on PET or PEN substrates with thicknesses of hundreds of micrometers while fragile indium tin oxide (ITO) transparent electrodes are normally used in the devices to achieve high efficiency.^[^ ^11^, ^12^ ^]^ Although the efficiency is relatively high, the mechanical flexibility and weight of the devices are generally limited by the brittleness of ITO and perovskite films, and the thick plastic substrate, which may not be able to meet the requirements for specific applications, like self‐powered bioelectronics, avionics, and wearable electronics where lightweight and ultrathin devices are highly desired.^[^ ^13^, ^14^ ^]^ Notably, the mechanical flexibility of a f‐PSC can be substantially improved by decreasing the thickness of the substrate to construct a ultrathin device.^[^ ^6^, ^15^ ^]^

Currently, ultrathin f‐PSCs show much lower efficiencies than that of a normal PSC due to their immature and complicated fabrication processes although many efforts have been devoted to fabrication techniques. Kaltenbrunner et al. reported an ultra‐thin f‐PSC fabricated on a 1.4 µm–thick PET substrate with a poly(3,4‐ethylenedioxythiophene)‐poly(styrenesulfonate) (PEDOT:PSS) transparent electrode, which exhibits a power‐per‐weight of 23Wg^−1^ and a PCE of 12%.^[^ ^16^ ^]^ Kang et al. used orthogonal silver nanowires (AgNWs) as the material of a bottom transparent electrode to fabricate an ultrathin f‐PSC with a PCE of 15.18% and a power‐per‐weight of 29.4 W g^−1^.^[^ ^17^ ^]^ The enhanced photovoltaic performance can be ascribed to the higher conductivity of AgNW than PEDOT:PSS. Regarding mechanical robustness, Lee et al. employed a synergistic effort of deceasing the substrate thickness to obtain a flexible device with a PCE of 17.03% and a high stability after 100 crumpling cycles.^[^ ^15^ ^]^ Recently, Wu et al. used Zr, Ti, and Ga‐doped indium oxide (ITGZO) as the bottom transparent electrode to prepare ultrathin f‐PSCs on 3 µm ‐thick parylene‐C substrates with a remarkable PCE of 20.2% and a power‐per‐weight of 30.3 W g^−1^.^[^ ^18^ ^]^ Recently, Hailegnaw et al. used quasi‐2D perovskite as an active layer to fabricate ultrathin PSCs with a champion PCE of 20.1% and a specific power up to 44 W g^−1^.^[^ ^19^ ^]^

Here, we report a highly flexible and efficient ultrathin perovskite solar cell, which is realized by the holistic optimization on perovskite films, transparent electrode, and substrate simultaneously. First, a 2D perovskite (PEA_2_PbI_4_) was introduced at grain boundaries of perovskite films as lubricants to release internal stress. As confirmed by in situ TEM characterization, applied stress on the films could be released by slippage or rotation of PEA_2_PbI_4_ 2D layers. Second, the conductivity of PEDOT:PSS transparent electrode was dramatically improved by doping it with sucralose. In addition, the doped PEDOT:PSS film shows much better mechanical flexibility than the pristine one while keeping comparable conductivity to ITO. Moreover, a stronger adhesion between PEDOT:PSS and perovskite layers has been formed due to the ultrasmooth morphology of PEDOT:PSS doped by sucralose. This not only leads to suppressed non‐radiative recombination but improves the mechanical stability of f‐PSCs. Third, an ultrathin PET substrate with a thickness of 1.4 um is employed to shift the neutral plane into the perovskite film to further enhance the mechanical durability. Based on the above strategies, lightweight and ultrathin perovskite solar cells are successfully prepared with the champion PCE of 21.44% and the maximum power‐per‐weight of 47.8 W g^−1^, which are the highest values for ultrathin f‐PSCs so far. Excellent mechanical flexibility and robustness have been achieved as the initial efficiency can be sustained after 1000 bending cycles at 0.5 mm curve radius. A stretchable PSC with the stretching ratio of up to 40% has been realized by laminating the ultrathin device on a pre‐strained flexible substrate. Moreover, the f‐PSC demonstrates excellent indoor photovoltaic performance by reaching a high efficiency of 36.25% under room light intensity. The high‐performance, lightweight, stretchable, and ultrathin f‐PSCs are expected to find niche applications in the future.

## 2 Results and Discussion

### 2.1 Incorporation of 2D Perovskite in 3D Perovskite

**Figure 1:**
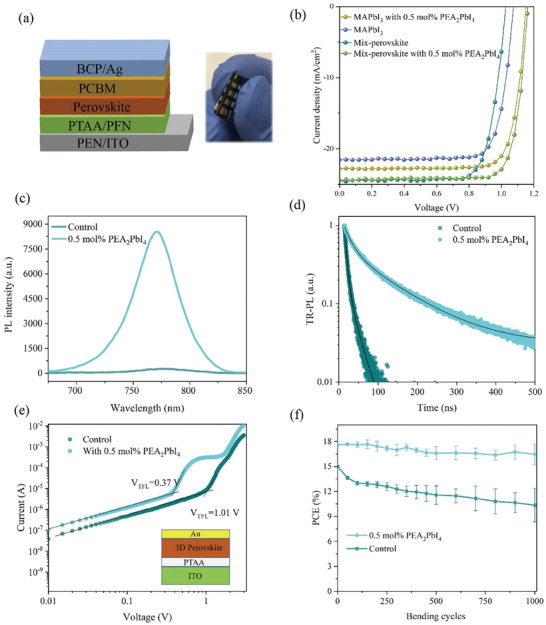
The effect of PEA_2_PbI_4_ additive on the performance of f‐PSCs and perovskite properties. a) schematic illustration of the device structure of a f‐PSC and a photograph of a flexible device. b) *J–V* curves of f‐PSCs with and without the addition of 0.5 mol% PEA_2_PbI_4_. c) Steady‐state PL and d) TR‐PL of perovskite films with and without PEA_2_PbI_4_. e) Current density‐voltage characteristics of hole‐only devices based on MAPbI_3_ films with and without PEA_2_PbI_4_. f) Efficiency evolution of f‐PSCs as functions of bending cycles.

In polycrystalline perovskite films, grain boundaries enriched with defects are the primary locations where cracks form under stress operation, deteriorating the efficiency and stability of the resultant flexible devices.^[^ ^20^, ^21^, ^22^, ^23^ ^]^ Hence, we introduced 2D perovskite PEA_2_PbI_4_ at the grain boundaries of 3D perovskites as a lubricant to suppress crack formation. **Figure** **1**a illustrates the device structure of a f‐PSCs where a commercial PEN/ITO substrate was used at the beginning. PEA_2_PbI_4_ was introduced as an additive in MAPbI_3_ perovskite in the devices. The detailed preparation process of perovskite films and devices can be found in supporting information. The photovoltaic performance of f‐PSCs is optimized by adjusting the amount of PEA_2_PbI_4_ in the perovskite precursor (see Figure S1 and Table S1, Supporting Information) and the device with 0.5 mol% PEA_2_PbI_4_ exhibits the best photovoltaic performance. Figure 1b shows the *J–V* curves of the champion f‐PSCs with and without the 2D perovskite. The control shows a PCE of 18.20% with a V_oc_ of 1.07 V, a J_sc_ of 21.56 mA cm^−2^ and an FF of 78.89%. The 2D modified device exhibits a PCE of 21.03% associated with a V_oc_ of 1.15 V, a J_sc_ of 22.79 mA cm^−2^, and an FF of 80.24%. To further improve the device efficiency, the mixed perovskite Cs_0.05_(FA_0.95_MA_0.05_)_0.95_Pb(I_0.95_Br_0.05_)_3_ was used as the active layer. The photovoltaic performance of flexible devices with different amounts of PEA_2_PbI_4_ is shown in Figure S2, Supporting Information. The detailed parameters were summarized in Table S2, Supporting Information. The optimal 0.5 mol% PEA_2_PbI_4_ was also introduced to improve the efficiency and mechanical stability. As shown in Figure 1b, with the addition of 0.5 mol% PEA_2_PbI_4_, the device PCE can be significantly improved from 19.58% to 22.90%, with V_oc_ increasing from 1.03 to 1.16 V, J_sc_ from 24.45 to 24.50 mA cm^−2^, and FF from 77.75 to 80.58%.

To elucidate the underlying mechanism of photovoltaic enhancement, steady‐state photoluminescence (PL) and time‐resolved PL of the perovskite films have been characterized, as shown in Figure 1c,d. It can be observed that the PL intensity was significantly increased with the addition of 0.5 mol% PEA_2_PbI_4_. The PL decay can be fitted by using a biexponential equation: Y = A_1_exp(‐t/τ_1_) + A_2_exp(‐t/τ_2_), where τ_1_ and τ_2_ correspond to the lifetimes of the fast and slow decay processes in the perovskite layer, respectively (se**e** Table S3, Supporting Information for the detailed parameters).The average decay time constant is calculated with the equation $\tau_{avg}=\frac{A_{1}\tau_{1}^{2}+A_{2}\tau_{2}^{2}}{A_{1}\tau_{1}+A_{2}\tau_{2}}$. The average carrier lifetime of MAPbI_3_ is substantially improved from 7.6 to 134.1 ns by the 2D perovskite. The enhanced PL intensity and carrier lifetime suggest that the introduction of 2D perovskite into the 3D perovskite film could significantly suppress non‐radiative recombination in the perovskite films.

To quantitatively evaluate the change of defect densities in perovskite films, hole‐only transport devices with the structure of ITO/PTAA/Perovskite/Au with and without the introduction of 0.5 mol% PEA_2_PbI_4_ are fabricated as shown in Figure 1e. The defect density for holes could be determined by the trap‐filled limit voltage (V_TFL_) according to the equation^[^ ^24^ ^]^

(1) $$N_{defects}=2\varepsilon \varepsilon_{0}V_{TFL}/eL^{2}$$

where *L* is the thickness of the perovskite film, ε and ε_0_ are the relative dielectric constant of MAPbI_3_ and the vacuum dielectric permittivity, respectively, and *e* is the elementary charge. The calculated defect densities (N_defects_) are 2.19 × 10^16^ cm^−3^ and 0.82 × 10^16^ cm^−3^ for the perovskite films without and with PEA_2_PbI_4_, respectively. The decreased defect density in the perovskite film after the incorporation of PEA_2_PbI_4_ is consistent with the results of PL characterization. Therefore, the enhanced photovoltaic performance, mainly the improved open circuit voltage, of the solar cells could be ascribed to defect passivation at grain boundaries by 2D perovskites.

Next, the flexible devices were characterized before and after multiple‐cycle bending under the bending curvature radius of 5.0 mm. As shown in Figure 1f, after bending for 1000 cycles, the f‐PSC with the addition of 2D perovskite PEA_2_PbI_4_ can maintain about 94% of its initial PCE, while the control device exhibits an obvious efficiency degradation to about 69% relatively. Hence, the introduction of PEA_2_PbI_4_ into the perovskite films can efficiently improve both the PCE and mechanical robustness of f‐PSCs.

### 2.2 Morphology and Mechanical Characteristics of Perovskite Thin Films

**Figure 2:**
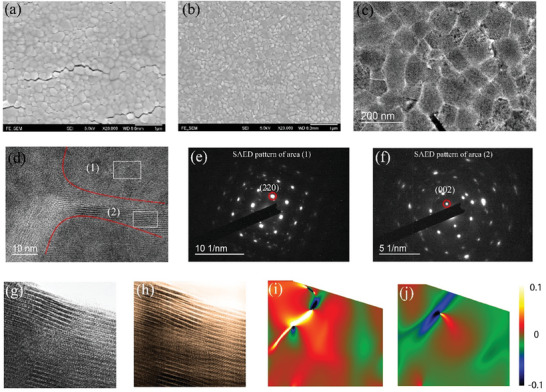
Microstructural images of perovskite films under strain. SEM images of a) a MAPbI_3_ film and b) a PEA_2_PbI_4_ ‐added film after 1000‐cyces bending. c) Low magnification TEM image of a PEA_2_PbI_4_ ‐added MAPbI_3_ film deposited on carbon support film. d) HRTEM image of grain boundaries of a PEA_2_PbI_4_ ‐added MAPbI_3_ film. SAED patterns of e) area 1 and f) area 2 of the perovskite film. g) Raw and filtered h) HRTEM image on the area close to the crack tip. i) The shear strain mapping and j) rotation angel mapping calculated from h).

To clarify the underlying mechanism of the enhanced bending durability of f‐PSCs by PEA_2_PbI_4_, the morphology evolutions of the perovskite films bent with different curvature radii were characterized under optical microscopy. Visible cracks can be found in MAPbI_3_ films after bending at a curvature radius of 3.0 mm and more cracks are generated at the bending radius of 1.5 mm (see Figure S3, Supporting Information). In comparison, PEA_2_PbI_4_ ‐added perovskite films show cracks at smaller bending radius (see Figure S4, Supporting Information) and the introduction of more PEA_2_PbI_4_ could make the perovskite films more bendable. **Figure** **2**a,b show SEM images of a MAPbI_3_ control film and a MAPbI_3_ film with 0.5 mol% PEA_2_PbI_4_ addition before and after bending for 1000 cycles under a radius of 3.0 mm. After bending, many cracks appear along grain boundaries in the control film while no obvious crack can be observed in the target film. These results suggest that the bending durability of the perovskite films is significantly improved by the introduction of PEA_2_PbI_4_.

The microstructure of perovskite films was then characterized to elucidate the underlying mechanism. The distribution of 2D perovskites in the MAPbI_3_ films can be observed under a transmission electron microscopy (TEM), as shown in Figure 2c. Figure 2d shows the high‐resolution TEM (HRTEM) image of grain boundaries of PEA_2_PbI_4_ ‐added MAPbI_3_ film, where lattice structures in grains and grain boundaries are obviously different, suggesting the different kinds of perovskite phases in the two regions. Selected‐area electron diffraction (SAED) pattern was characterized on area (1) and (2) corresponding to grain and grain boundary regions, respectively, to find the corresponding lattice structures, as shown in Figure 2e,f. The diffraction spot marked by a red circle in area (1) can be referred to (220) plane of MAPbI_3_ due to the matched interplane distance of 0.31 nm, while the diffraction spot marked by a red circle in area (2) can be indexed as (002) plane of PEA_2_PbI_4_ with the interplane spacing of 0.75 nm, demonstrating that the material at the grain boundary is 2D perovskite PEA_2_PbI_4_. The TEM images of the MAPbI_3_ perovskite films demonstrate that 2D perovskites are mainly located at the grain boundaries of perovskite films. It is expected that the 2D phase between perovskite grains can release strain by the reversible sliding or rotation between neighboring layers, as illustrated in Figure S5, Supporting Information.^[^ ^25^, ^26^, ^27^ ^]^ For the pristine MAPbI_3_ film, it can be observed that there are some holes or amorphous structure at the grain boundaries (see Figure S6, Supporting Information), which is unfavorable for device performance. Hence, the 2D perovskite can fill the holes and replace the amorphous phase at grain boundaries of 3D perovskites.

To investigate how 2D perovskites can release strain at grain boundaries, in situ strain was applied on a perovskite film via tearing the underlying carbon film with electron beam under TEM. This method is usually used to study the lattice change of nanomaterials under strain.^[^ ^28^, ^29^ ^]^ It can be found that cracks are formed along the grain boundaries, which suggests that strain in the perovskite film tends to be released by forming cracks along the grain boundaries (see Figure S7, Supporting Information). Since the crack tip is the stress concentrating area, HRTEM observation is conducted on this area. Figure 2g and h show the raw and filtered HRTEM image of a local area close to the crack tip, respectively. Deformed 2D crystal lattice close to the crack tip can be clearly observed from the geometric phase analysis (GPA) displayed in Figure 2i,j, which can be attributed to the factor that the elastic modulus (shear modulus e12 and normal modulus e33) of the 2D perovskite is much smaller than 3D perovskite. Notably, the weak van der Waals (vdW) interactions between 2D perovskite layers can allow lattice deformation and even slippage on the 2D plane.^[^ ^27^ ^]^ The strain‐concentrated zones can reach a local ultrahigh shear strain level of about ±10%, indicating a high flexibility of the 2D perovskite phase. Therefore, the flexible 2D perovskite located at the grain boundaries provides abundant pathways for strain release in the 2D/3D mixed perovskites. In addition, DFT calculation shows that PEA_2_PbI_4_ has much lower Young's modulus along out‐of‐plane orientation, which is due to the presence of weak van der Waals interaction and organic spacer. It can enable the grain boundaries of MAPbI_3_ films to be more deformable by introducing 2D PEA_2_PbI_4_ with much smaller Young's modulus along out‐of‐plane direction (see Figures S8 and S9 and Note S1, Supporting Information).

### 2.3 Highly Conductive PEDOT:PSS Electrodes for f‐PSCs

**Figure 3:**
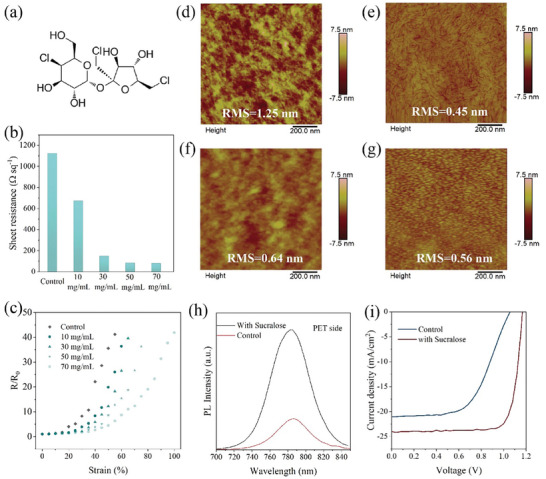
Characterization of highly conductive and flexible PEDOT:PSS doped with sucralose. a) The molecular structure of sucralose. b) Sheet resistance of PEDOT:PSS film with different concentration of sucralose. c) Resistant change of PEDOT:PSS films doped with sucralose under different compression strain. Atomic force microscopy images of PEDOT:PSS films d) without and e) with sucralose. Atomic force microscopy images of PTAA films deposited on PEDOT:PSS films f) without and g) with sucralose. h) Steady‐state PL spectra of perovskite films deposited on PEDOT:PSS films with and without sucralose. i) *J–V* curves of f‐PSCs based on mixed perovskite.

PEDOT:PSS is a conductive material popularly used in flexible electronics due to its intrinsically flexible and conductive nature.^[^ ^30^ ^]^ However, normal PEDOT:PSS cannot meet the requirement of high conductivity for transparent electrodes in f‐PSCs. To improve its conductivity, PEDOT:PSS (PH1000) was doped by sucralose for the first time with different amounts, as shown in **Figure** **3**a. Sucralose is known to be an low cost artificial sweetener. Here, it can be used as a plasticizer in PEDOT:PSS because it can form hydrogen bonds with the −OH groups of PSS and increase the flexibility of the polymer chains. The formation of hydrogen bond is confirmed by Fourier‐transform infrared spectroscopy (FTIR) measurement as shown in Figure S10 and Note S2, Supporting Information. Interestingly, the addition of sucralose leads to negligible change on the transparency of PEDOT:PSS films, which is higher than 90% in the visible region (as shown in Figure S11, Supporting Information). Figure 3b shows the sheet resistance of PEDOT:PSS films based on different doping amounts of sucralose. The sheet resistance of PEDOT:PSS film can be significantly decreased from about 1200 to 50 Ω sq^−1^ when the addition concentration of sucralose is increased to 50 mg mL^−1^ (see Figure S12, Supporting Information). The improved conductivity is attributed to the higher content of PEDOT in the film, according to X‐ray photoelectron spectroscopy (XPS) characterization (see Figure S13 and Note S3, Supporting Information), and the formation of nanofibers of PEDOT with introduction of sucralose. Then, PEDOT:PSS thin films were spin coated on the PDMS substrates to measure their electrical stability during stretching processes. As shown in Figure 3c, the PEDOT:PSS films can sustain higher strain at higher sucralose concentrations. PEDOT:PSS films with additions of 50 or 70mg mL^−1^ of sucralose can maintain their high conductivity at a strain up to 40%.

PEDOT:PSS thin films have been observed under optical microscopy with 0% and 40% strain. As shown in Figure S14, Supporting Information, many cracks appeared in the pristine PEDOT:PSS thin film under 40% tensile strain. In comparison, several wrinkles, instead of cracks, can be observed in the film with sucralose under the same tensile strain. The atomic force microscopy (AFM) characterization was employed to elucidate the morphology change of PEDOT:PSS doped by sucralose. As shown in Figure 3d, the pristine PEDOT:PSS film exhibited amorphous morphology of large‐domain PEDOT:PSS aggregates, which leads to the low conductivity due to the insulating property of PSS.^[^ ^31^ ^]^ In comparison, the sucralose doped PEDOT:PSS film showed absolutely different morphology where a phase separation was achieved by the formation of ordered stacking PEDOT nanofibrils, which can lead to higher conductivity and mechanical stretchability of the PEDOT:PSS film.^[^ ^30^, ^32^ ^]^ To further confirm the phase change and distribution of PEDOT:PSS with sucralose demonstrated in AFM characterization, we have conducted Raman characterization on PEDOT:PSS films with and without sucralose. As shown in Figure S15, Supporting Information, C_α_ = Cβ vibration peaks (≈1429 cm^−1^) in the Raman spectroscopy shows a red‐shift and narrower in width in the PEDOT:PSS films with addition of sucralose. This indicates that a higher proportion of the benzoid moieties in PEDOT are converted to the quinoid structure, which results in a more planar backbone, consistent with the formation of nanofibers of PEDOT shown in the AFM images.^[^ ^30^, ^31^ ^]^

In addition, the roughness of PEDOT:PSS film is significantly reduced from 1.25 to 0.45 nm with the addition of sucralose. In comparison with other additives, sucralose can result in the highest improvements on the conductivity and morphology control based on spin‐coating films in photovoltaic applications (Table S4, Supporting Information), which is critical to the future application of PEDOT:PSS in ultraflexible electronics. This ultralow roughness of PEDOT:PSS is highly important for the deposition of continuous PTAA HTL with very thin thickness, which can guarantee the hole transporting and electron blocking functions. As shown in Figure 3f, the PTAA film deposited on the pristine PEDOT:PSS control film showed large aggregates with a discontinuous morphology, while the PTAA on the target PEDOT:PSS demonstrated a compact and smooth morphology, which is more favorable for hole transfer in a PSC. Besides, the PEDOT:PSS electrodes with ultrasmooth morphology can enable a compact interface between PTAA and perovskite layers. Figure S16, Supporting Information showed the distribution of perovskite films after being peeled off from two different PEDOT:PSS substrates. It can be clearly observed that some parts of the perovskite film was left on the doped PEDOT:PSS while the perovskite on pristine PEDOT:PSS was completely removed, which indicates the formation of a more compact interface in the former. This compact interface can suppress non‐radiative recombination at the interface, which is confirmed by a much higher PL intensity (4.45 times) for the target sample (Figure 3h).

Then, PEDOT:PSS thin films doped with sucralose were used as the bottom electrodes in f‐PSCs. All flexible devices have used PEA_2_PbI_4_ ‐modified perovskites as active layers. Figure S17, Supporting Information shows the *J–V* curves of f‐PSCs based on PEDOT:PSS electrode with different amounts of sucralose. The device based on the pristine PEDOT:PSS and MAPbI_3_ perovskite can only achieve a low PCE of 11.23% with a V_oc_ of 1.04 V, a J_sc_ of 19.46 mA cm^−2^, and an FF of 55.49%. With the addition of 50 mg mL^−1^ sucralose in PEDOT:PSS, the device PCE can be significantly increased to 20.07% with a V_oc_ of 1.13 V, a J_sc_ of 23.24 mA cm^−2^ and an FF of 76.42%. Therefore, the enhanced conductivity of PEDOT:PSS electrode and perovskite/PEDOT:PSS interface contact by sucralose doping makes great contribution to the overall improvement of as‐prepared f‐PSCs. To further improve the device performance, mixed perovskites Cs_0.05_(FA_0.95_MA_0.05_)_0.95_Pb(I_0.95_Br_0.05_)_3_ have been introduced in the f‐PSCs. The PCE of the f‐PSC was increased from 12.67% to 22.43% after the addition of 50 mg mL^−1^ sucralose in the PEDOT:PSS electrode (Figure 3i). The control device shows a V_oc_ of 1.05 V, a J_sc_ of 21.06 mA cm^−2^ and an FF of 56.98%. The optimized device shows a V_oc_ of 1.16 V, a J_sc_ of 23.99 mA cm^−2^ and an FF of 80.6%. The EQE curves of the control and optimized device are shown in Figures S18 and S19, Supporting Information, respectively, which exhibits a reasonable integrated J_sc_ of 20.0 and 23.6 mA cm^−2^, respectively.

### 2.4 Ultrathin f‐PSCs with High Photovoltaic Performance and Mechanical Durability

**Figure 4:**
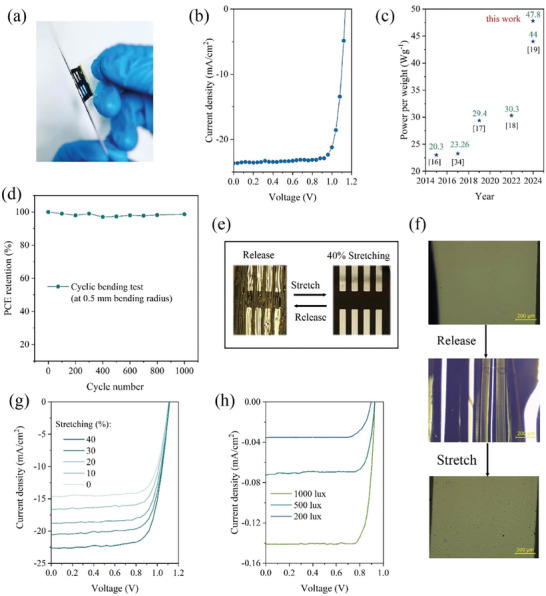
Characterization of ultrathin f‐PSCs. a) A photograph of an ultrathin f‐PSC wrapping upon a needle. b) *J–V* curves of an ultrathin f‐PSC. c) Summary of power‐per‐weight of typical ultrathin f‐PSCs. d) PCE evolution of a f‐PSC under bending tests at 0.5 mm radius for 1000 cycles. e) Photographs of an ultrathin f‐PSC before and after strain release. f) Optical microscopy images of a stretchable f‐PSC under stretched and released statuses. g) *J–V* curves of an ultrathin f‐PSC under different stretching ratios. h) *J–V* curves of an ultrathin f‐PSC under different indoor light intensity.

To further improve the mechanical flexibility of f‐PSCs, PET substrates with an ultrathin thickness of 1.4 µm have been used to position the neutral plane, where the net strain and stress is zero, closer to or within the perovskite films, and decease the total device thickness.^[^ ^33^ ^]^ The use of PET substrates with a thickness of 1.4 µm could position the neutral plane at perovskite layer, which could significantly improve the mechanical flexibility of f‐PSCs (See Figure S20, TableS5 and Note S4, Supporting Information). **Figure** **4**a shows a photograph of an ultrathin f‐PSC wrapping up a needle, which indicates the ultra‐flexible property of the device. Figure 4b shows the *J–V* curve of a champion device, which shows a PCE of 21.44% as well as a V_oc_ of 1.134 V, a J_sc_ of 23.65 mA cm^−2^, an FF of 79.94%. The mean PCE of ten devices is about 19.77 ± 1.5% (See Figure S21, Supporting Information). The quality density of our devices is about 4.48 gm^−2^ (see Figure S22, Supporting Information). The highest power‐per‐weight of the device is estimated to be 47.8 W g^−1^, which is a record value for ultrathin f‐PSCs so far (Figure 4c and Table S6, Supporting Information). The bending stability is also measured under a bending radius of 0.5 mm. The ultrathin f‐PSC can keep the initial PCE after 1000 bending cycles, as shown in Figure 4d.

Stretchable PSCs can be realized by laminating the flexible ultrathin f‐PSCs on pre‐strained PDMS substrates. The PET bottom surface of the device can be easily attached onto the PDMS surface due to van der Waals interaction. Figure 4e shows photographs of a f‐PSC attached to a pre‐strained (40% tensile strain) PDMS substrate before and after the release of strain. The stretchable f‐PSC shows stable performance without visible damage on the device surface during the stretching tests. Under optical microscopy, several wrinkles were observed on the surface of the f‐PSCs after strain release and they totally disappeared when the device was stretched back for 40% to its length, as shown in Figure 4f. Figure 4g shows *J–V* curves of a f‐PSC under different stretching ratios. V_oc_ and FF of the device have not been changed significantly while J_sc_ increases with the increase of stretching ratio as the illuminated active area increases accordingly. The device has been characterized under stretching tests for 1000 times and shows little change in its photovoltaic performance (see Figure S23, Supporting Information).

Considering the great potential of a PSC for indoor applications, indoor photovoltaic performance of the ultrathin f‐PSC is characterized, as shown in Figure 4h. The PCEs of 36.25%, 33.42% and 32.42% can be achieved under 1000 lux, 500 lux and 200 lux white light, respectively (Figure S24, Supporting Information). The corresponding photovoltaic parameters can be found in Table S7, Supporting Information. Notably, this is the first demonstration of ultrathin f‐PSCs for indoor light energy harvesting. Considering its ultrahigh power‐per‐weight value, the device is expected to find many niche applications such as wearable electronics and IOT.

## 3 Conclusion

In summary, ultrathin f‐PSCs with high mechanical flexibility and power conversion efficiencies have been fabricated with three strategies for material and device optimization. First, the introduction of a 2D perovskite PEA_2_PbI_4_ in 3D perovskite phases can significantly improve the mechanical flexibility and carrier lifetime of the perovskite active layer. Second, intrinsically flexible PEDOT:PSS film shows dramatically improved flexibility and conductivity after the addition of sucralose, which acts as the transparent electrode of the device. Third, 1.4 µm‐thick PET substrate is used in the devices to shift the neutral plane within the perovskite film, further improving the mechanical robustness of the device. The ultrathin f‐PSCs have achieved a high efficiency over 21% and a record power‐per‐weight of 47.8 W g^−1^ for f‐PSCs. A stretchable device based on the ultrathin f‐PSC shows excellent device stability under tensile strain for up to 40%. In addition, the device can achieve a high PCE of 36.25% under 1000 lux light, showing great potential in indoor photovoltaic applications.

## Conflict of Interest

The authors declare no conflict of interest.

## Supporting information



Supporting Information

## Data Availability

The data that support the findings of this study are available from the corresponding author upon reasonable request.
