# Supplementary material for: Holistic Optimization toward Ultrathin Flexible Perovskite Solar Cells with High Efficiency and Mechanical Robustness
Source: Adv Sci (Weinh). 2025 Jun 2;12(27):2415372. doi: 10.1002/advs.202415372 (PMC12279163; doi:10.1002/advs.202415372)
Supplement: Supplementary file 1 — Supporting Information [file ADVS-12-2415372-s001.docx]

**Holistic optimization toward ultrathin flexible perovskite solar cells with high efficiency and mechanical robustness**

Guanqi Tang, Fangyuan Zheng, Jiajun Song, Qidong Tai, Jiong Zhao, Feng Yan*

G. Q. Tang

Research Institute of Frontier Science, Southwest Jiaotong University, Chengdu 610031, P. R. China

G. Q. Tang, F. Y. Zheng, J. J. Song, J. Zhao, F. Yan

Department of Applied Physics, Research Center for Organic Electronics, The Hong Kong Polytechnic University, Hung Hom, Kowloon, Hong Kong 999077, P. R. China

E-mail: [apafyan@polyu.edu.hk](mailto:apafyan@polyu.edu.hk)

Q. D. Tai

The Institute of Technological Sciences, Wuhan University, Wuhan 430072, P. R. China

F. Yan

Research Institute of Intelligent Wearable Systems, The Hong Kong Polytechnic University, Hung Hom, Kowloon, Hong Kong 999077, P. R. China.

**Experimental section:**

**Preparation of f-PSCs**: Commercial PEN/ITO substrates were used as received by attaching to a glass to prepare devices. PTAA (Sigma) dissolved in toluene (2.0 mg/mL^−1^) was spin-coated on the PEN/ITO substrates at a spin rate of 5000 rpm for 40 s. The films were subsequently annealed on a hotplate at 100 °C for 10 min. PFN was spin-coated on the PTAA film to increase the hydrophilicity of PTAA film to facilitate the deposition of perovskite film. The perovskite films were prepared by spin-coating the perovskite precursor solution containing CH_3_NH_3_I (Dyesol): 159 mg; PbI_2_ (Alfa, 99.99%): 461 mg; DMF (Sigma, anhydrous, 99.9%): 600 mg; and DMSO (Sigma, anhydrous, 99.9%): 78 mg at 4000 rpm for 30 s. During the spin coating process, 0.5 mL of diethyl ether (Sigma, anhydrous, 99.7%) was slowly dripped on the rotating substrate in 10 s after starting. All perovskite films were annealed at 100 °C for 20 min. The mixed perovskite was prepared by mixing 1.2 M FAPbI_3_ and MAPbBr_3_ perovskite solution in DMF:DMSO (4:1 volume) in a ratio of 95:5. The 1.2 M FAPbI_3_ solution was thereby prepared by dissolving FAI (722 mg) and PbI2 (2130 mg) in 2.8 mL DMF and 0.7 mL DMSO that contains a 10 molar % excess of PbI_2_. The 1.2 M MAPbBr3 solution was made by dissolving MABr (470 mg) and PbBr_2_ (1696 mg) in 2.8 mL DMF and 0.7 mL DMSO that contains a 10 molar % excess of PbBr_2_. 40 uL of 1.5 M CsI solution in DMSO (389 mg CsI in 1 mL DMSO) was mixed with 960 uL of the above mixture of FAPbI_3_ and MAPbBr_3_. The PEA_2_PbI_4_ precursor (DMF) was prepared with equal molar weight to the MAPbI_3_ precursor. Different amount of PEA_2_PbI_4_ precursor is mixed with MAPbI_3_ or mixed perovskite precursor to prepare perovskite films with 2D perovskites. Next, electron transport layers (ETLs) were prepared by spin coating a solution of PCBM (Nano-C) in chlorobenzene (20 mg/ml) at 3000 rpm for 40 s, followed by a thermal annealing at 90 *°*C for 20 min to crystalize PCBM. Bathocuproine (BCP, Sigma, 96%) dissolved in methanol (0.5 mg/ml) was spin-coated on the PCBM films at 4500 rpm for 30 s. Finally, devices were completed with the evaporation of silver (Ag) top electrodes. The ultrathin f-PSCs were prepared on an ultrathin PET substrate attached on PDMS/glass via van der Waals interaction. The fabrication process is same as the rigid counterpart. After finished, the devices can be peeled off from the PDMS substate easily due to the week interaction.

**Material and device characterization:** Scanning electron microscopy (SEM) images of MoS_2_ and perovskite thin films were obtained under a Hitachi S-4300 microscope. The TEM images of MoS_2_ and perovskite were conducted using JEOL JEM-2100F TEM/STEM operated at 200 kV. X-ray diffraction (XRD) measurement was performed using a Rigaku SmartLab X-ray Diffractometer operating at room temperature. Time-resolved photoluminescence (PL) measurements of the samples were carried out by using an Edinburgh FLSP920 fluorescence spectrophotometer. A 485-nm laser was used as an excitation light source.

The current density versus voltage (J-V) characteristics of the PSCs were measured by using a Keithley 2400 source meter under the illumination of an AM 1.5 solar simulator with a light intensity of 100 mW/cm^2^ (Newport 91160, 300W). The light intensity was calibrated with a standard silicon solar cell. The external quantum efficiencies (EQEs) of the PSCs were measured with a standard test system, including a xenon lamp (Oriel 66,02, 300W), a Si detector (Oriel 76175_71580), a monochromator (Newport 66902) and a dual channel power meter (Newport 2931_C). The impedance characterization was performed by ZAHNER ENNIUM electrochemical workstation.


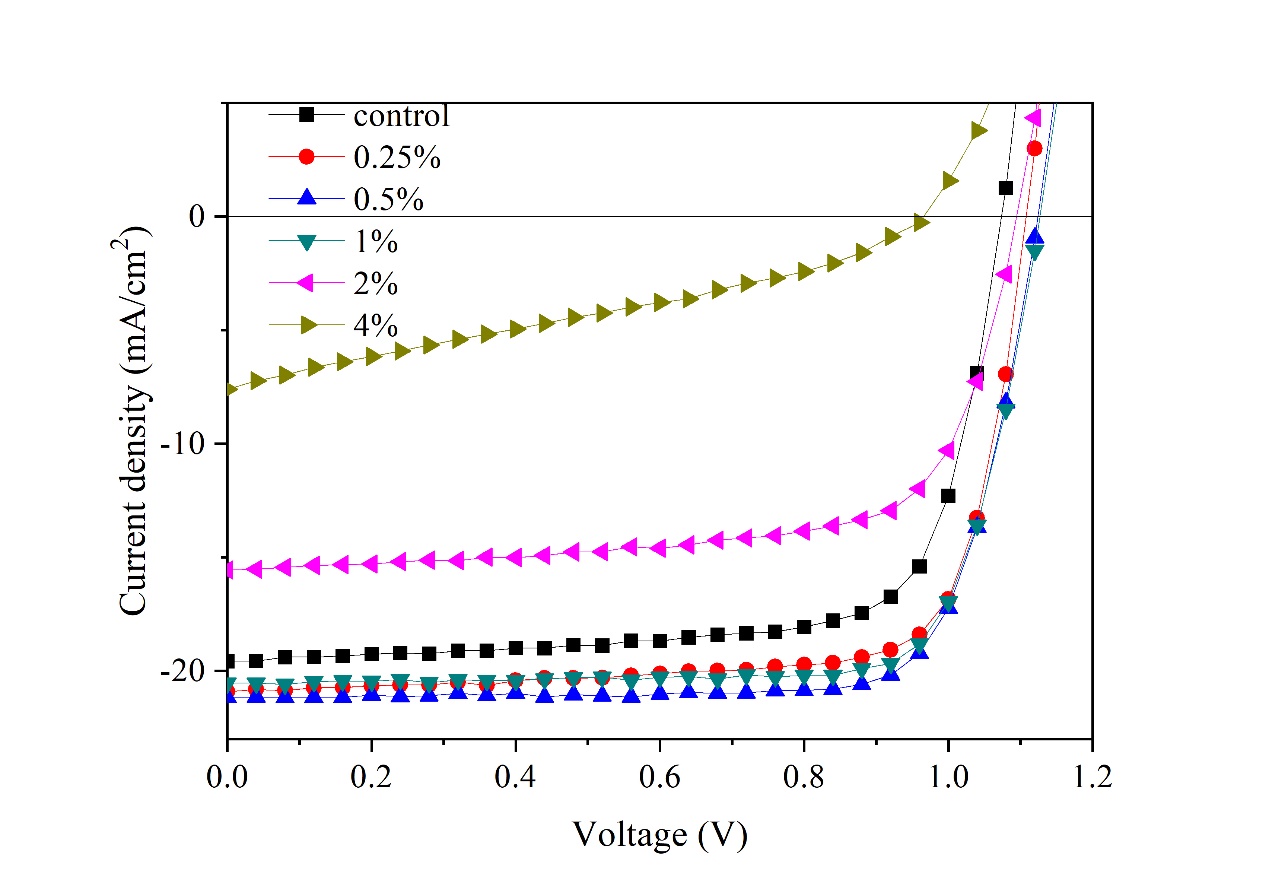


**Figure S1**. The J-V curves of FPSCs based on MAPbI_3_ and MAPbI_3_ with different amounts of PEA_2_PbI_4_.


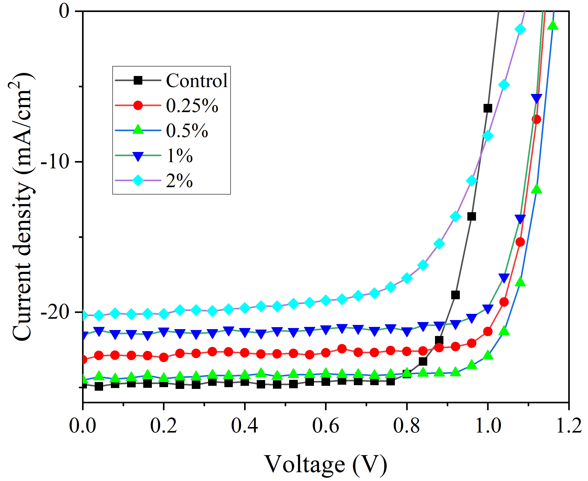


**Figure S2**. The J-V curves of FPSCs based on mixed perovskite with different amounts of PEA_2_PbI_4_.


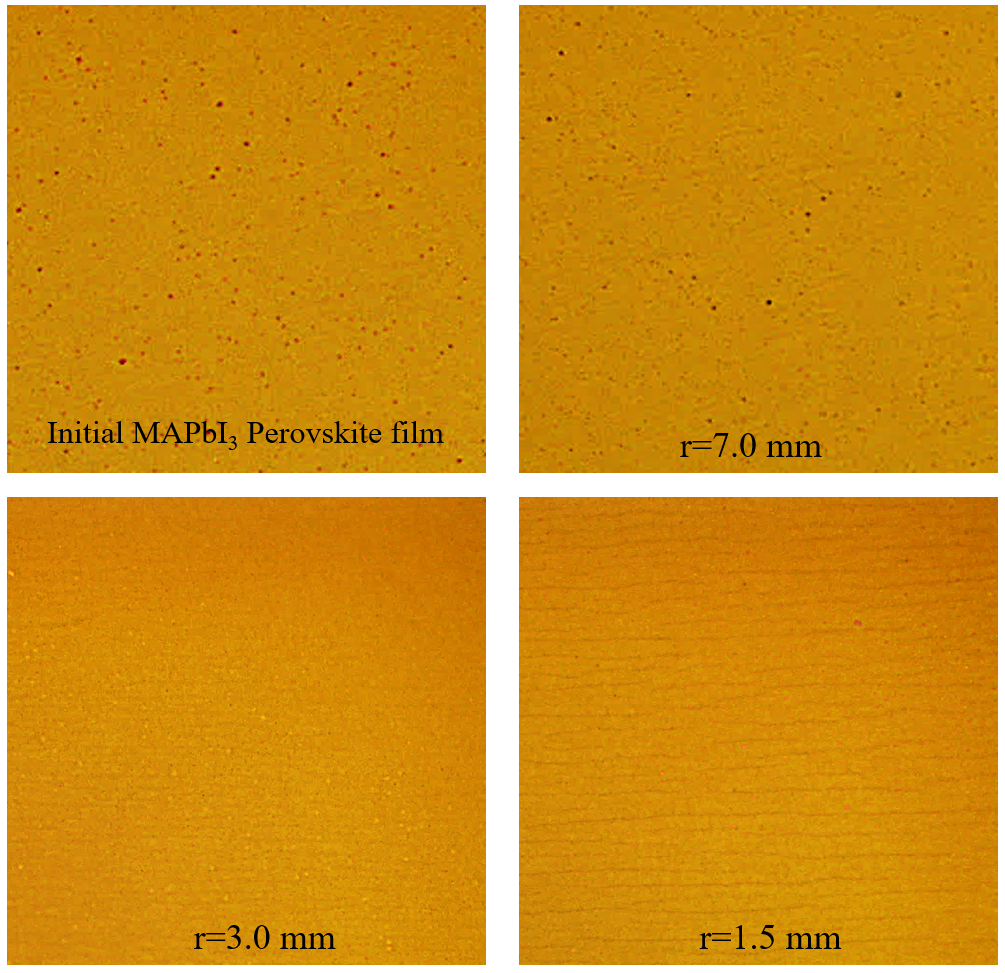


**Figure S3**. The bending evolution of MAPbI_3_ films along different radius under optical microscope characterization.


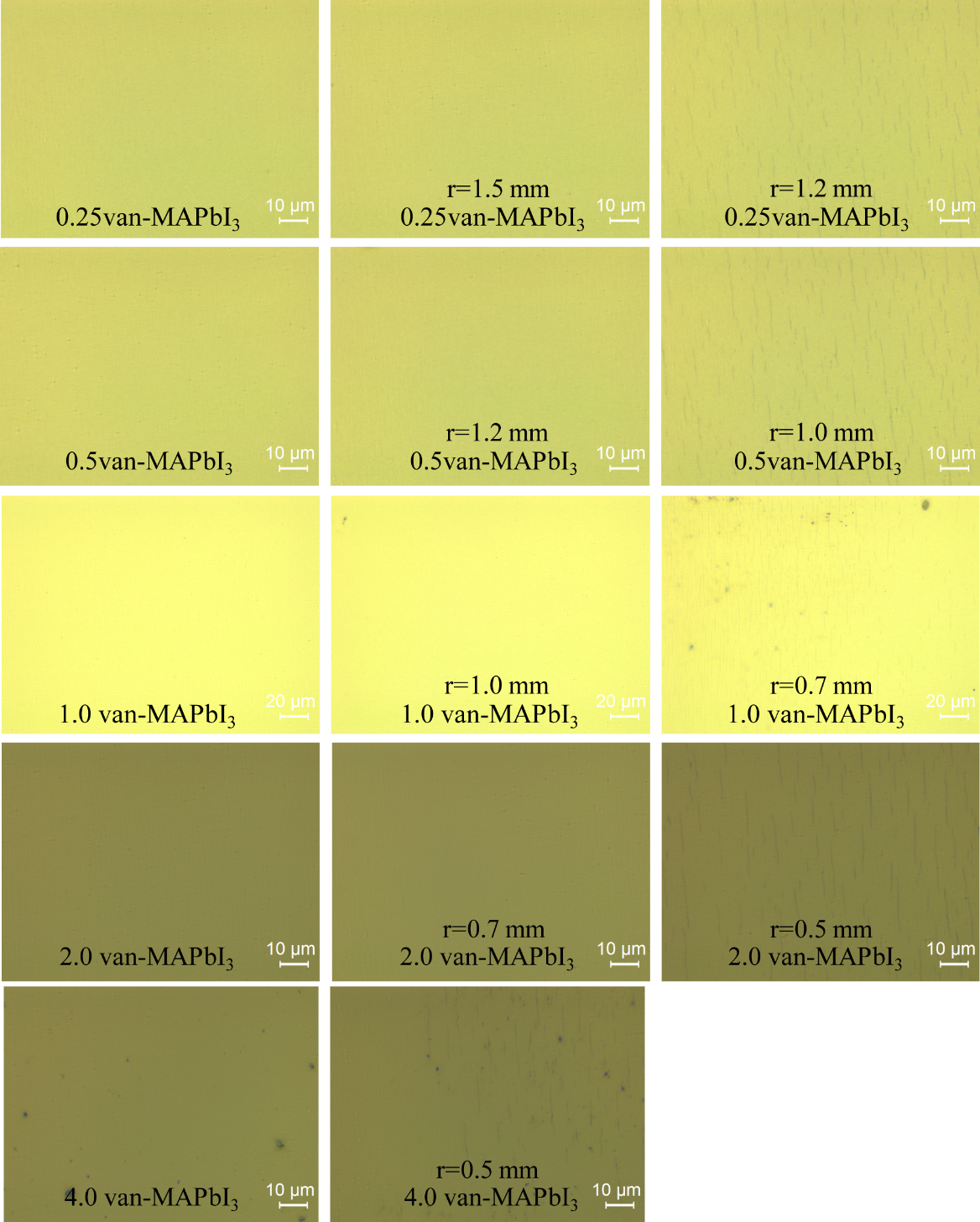


**Figure S4**. The optical images of 2D modulated MAPbI_3_ with different amount of PEA2PbI4 under different bending radius.


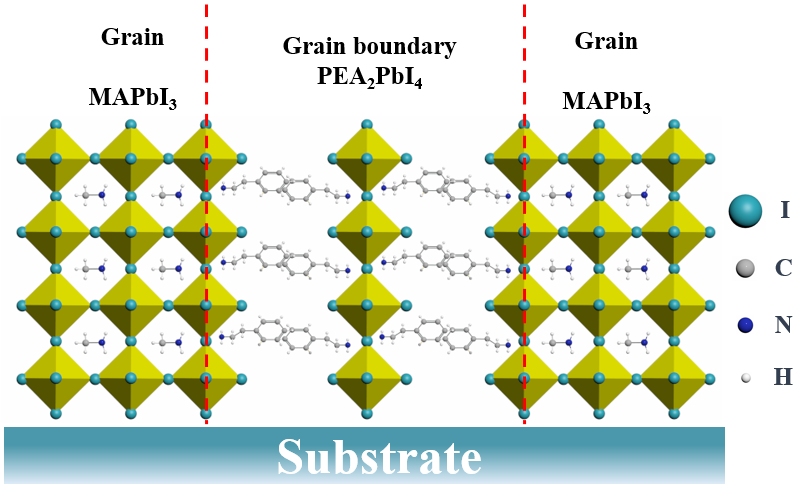


**Figure S5**. Illustration of PEA_2_PbI_4_ locating at grain boundaries of MAPbI_3_ perovskite film.


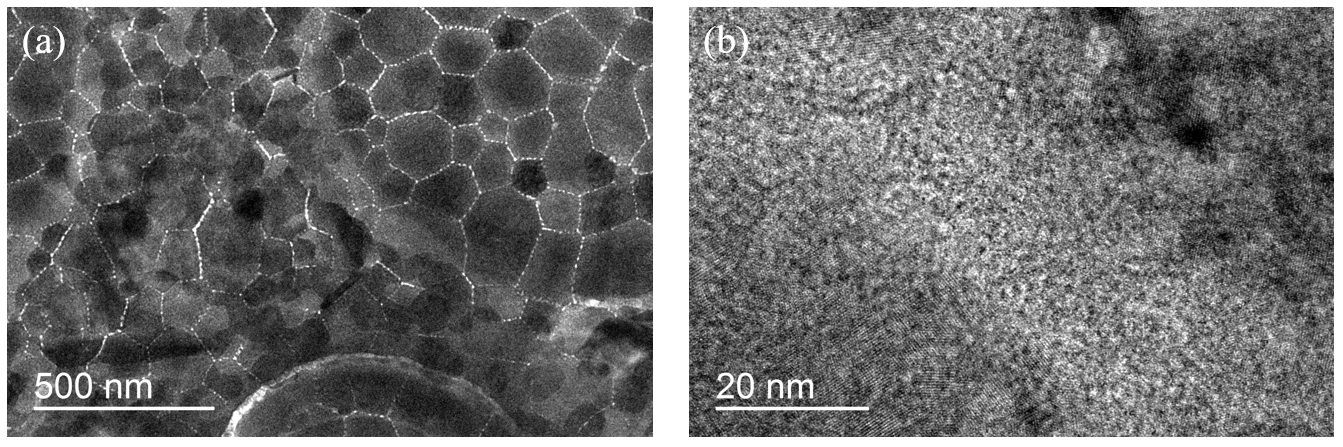


**Figure S6**. TEM and HRTEM images of grain boundaries of MAPbI_3_ film.


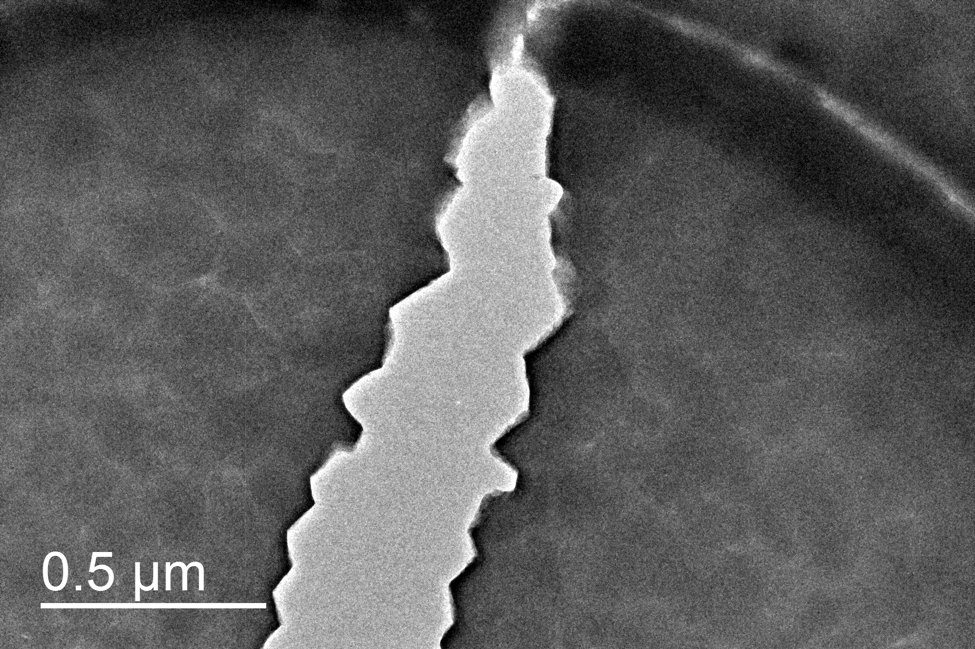


**Figure S7**. The TEM image of cracks in van-MAPbI_3_ film split by the underlying carbon film.

**
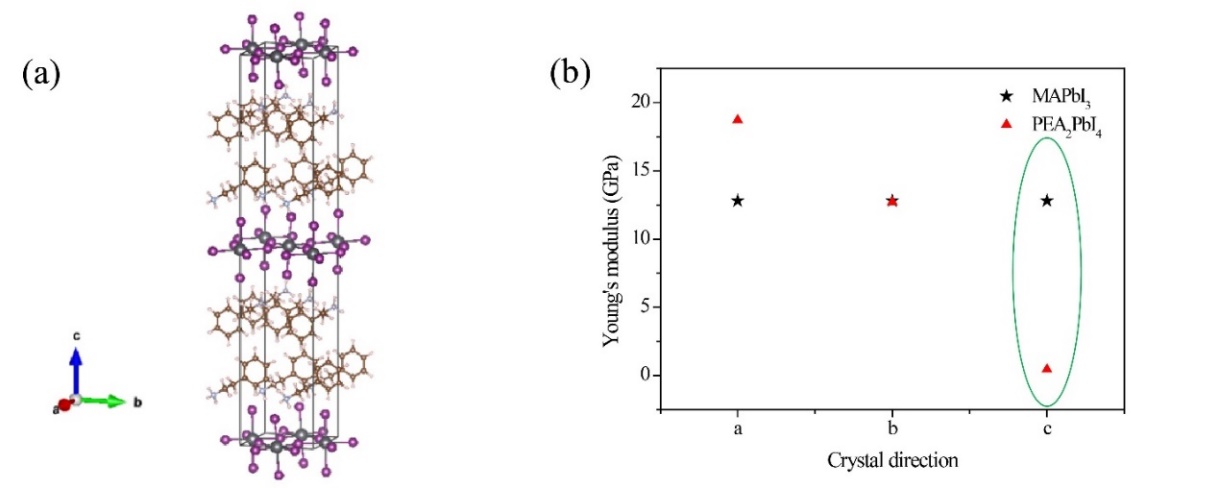
**

**Figure S8**. a) The crystal structure of PEA_2_PbI_4_. b) The DFT calculated Young’s modulus of MAPbI_3_ and PEA_2_PbI_4_.


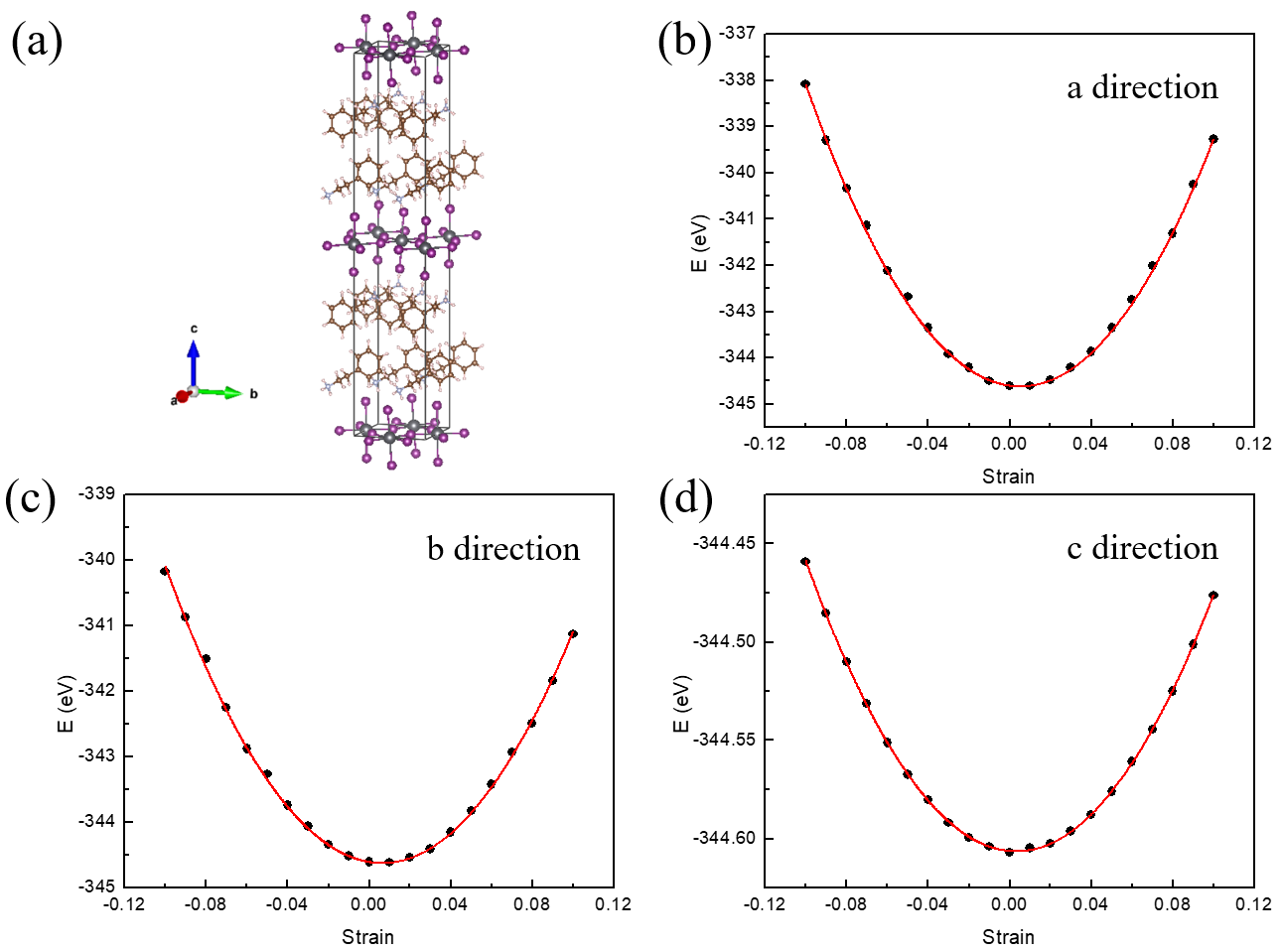


**Figure S9**. Simulation of PEA_2_PbI_4_ perovskite. a) The supercell structure used for simulation. b-c) The potential energy against strain for 2D perovskite along a, b, and c direction predicted by DFT calculations.

**Note 1. The DFT calculation on the Young’s modulus of MAPbI3 and PEA2PbI4.**

The theoretical calculations of the Young’s modulus on 3D and 2D perovskite materials have also been conducted to deeply understand the mechanical properties. The lower value of Young’s modulus could suggest higher deformability of the material. **Figure S8a** shows the crystal structure of PEA_2_PbI_4_. The DFT calculation is employed to determine the Young’s modulus values of MAPbI_3_ and PEA_2_PbI_4_ perovskites (see **Figure S9**). As shown in **Figure S8b,** the in-plane Young’s modulus of PEA_2_PbI_4_ along *a* and *b* directions are calculated to be 18.75 GPa and 12.71 GPa, respectively. These values are comparable to that of MAPbI_3_ (APL Materials 2014, 2, 081801). The out-of-plane Young’s modulus along *c* direction is calculated to be 0.44 GPa, which is substantially lower than that of 3D MAPbI_3_ perovskite. This much lower value for PEA_2_PbI_4_ along c direction (out-of-plane orientation) is due to the presence of weak van der Waals interaction and organic spacer. The result of theoretical calculation suggests that it can enable the grain boundaries of MAPbI_3_ films to be more deformable by introducing 2D PEA_2_PbI_4_ with much smaller Young’s modulus along out-of-plane direction.


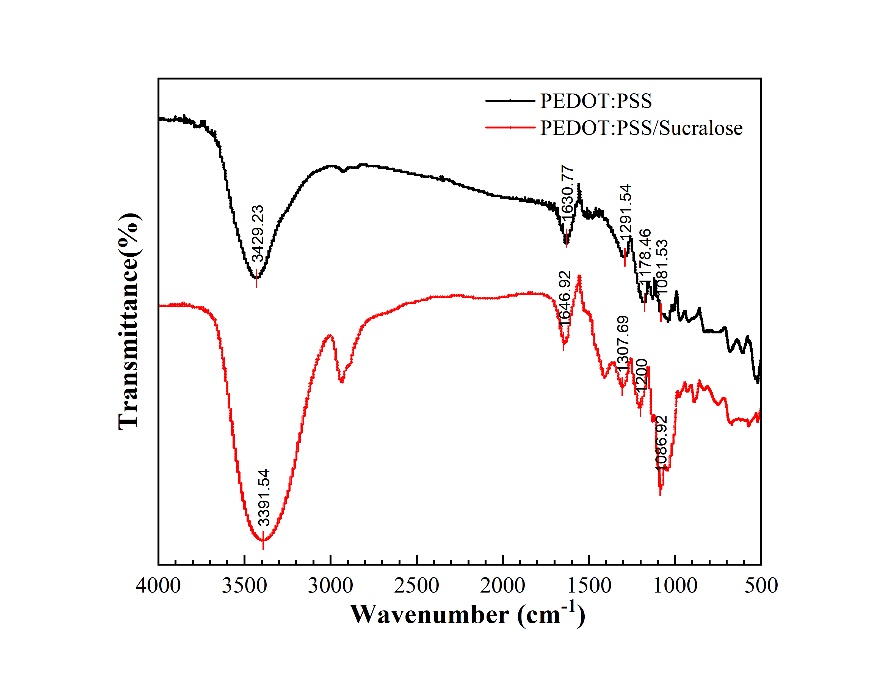


**Figure S10**. Fourier-transform infrared spectroscopy (FTIR) spectra of PEDOT:PSS with and without sucralose.

**Note 2. The FTIR analysis of the formation of hydrogen bonds between PEDOT:PSS and sucralose**

To confirm the hydrogen bond between sucralose and PEDOT:PSS, the FTIR spectroscopy is conducted on PEDOT:PSS and PEDOT:PSS/Sucralose films. As shown in Figure S8, the band at 3429 cm^-1^ is assigned to -OH stretching from PSS of PEDOT:PSS (Mater. Adv., 2023, 4, 4732-4743). This band was shifted to 3391 with addition of sucralose indicating the interaction of hydrogen bond between PSS and sucralose. Moreover, the bands at 1178 cm^-1^ and 1291 cm^-1^, which are assigned to S=O stretching of PSS, were shifted to 1200 cm^-1^ and 1307 cm^-1^, respectively (ACS Appl. Mater. Interfaces 2014, 6, 17792-17803). This further confirmed the interaction of hydrogen bond between PSS and sucralose. The hydrogen bond between PSS and sucralose would result in screen effect which could decrease the Coulomb interaction to facilitate the phase separation between PEDOT and PSS (Adv. Sci. 2019, 6, 1900813).


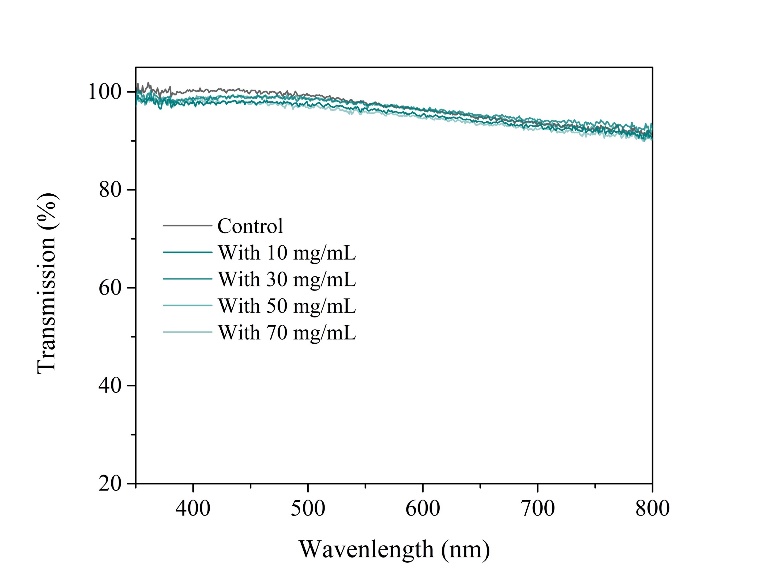


**Figure S11**. The transmission of PEDOT:PSS films with different sucralose concentration.


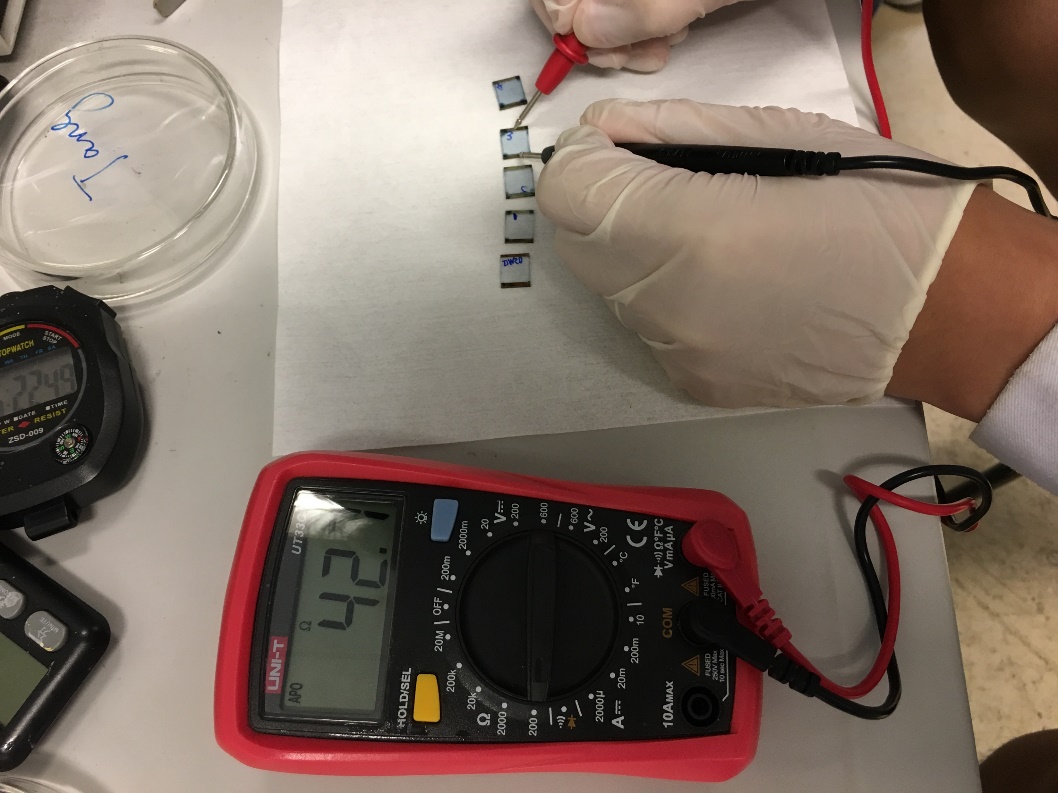


**Figure S12**. The resistance of PEDOT:PSS film with 50 mg/mL sucralose measured by an ohmmeter. The sheet resistance of a PEDOT:PSS film with square area was measured via two gold electrodes at opposite sides by an ohmmeter.


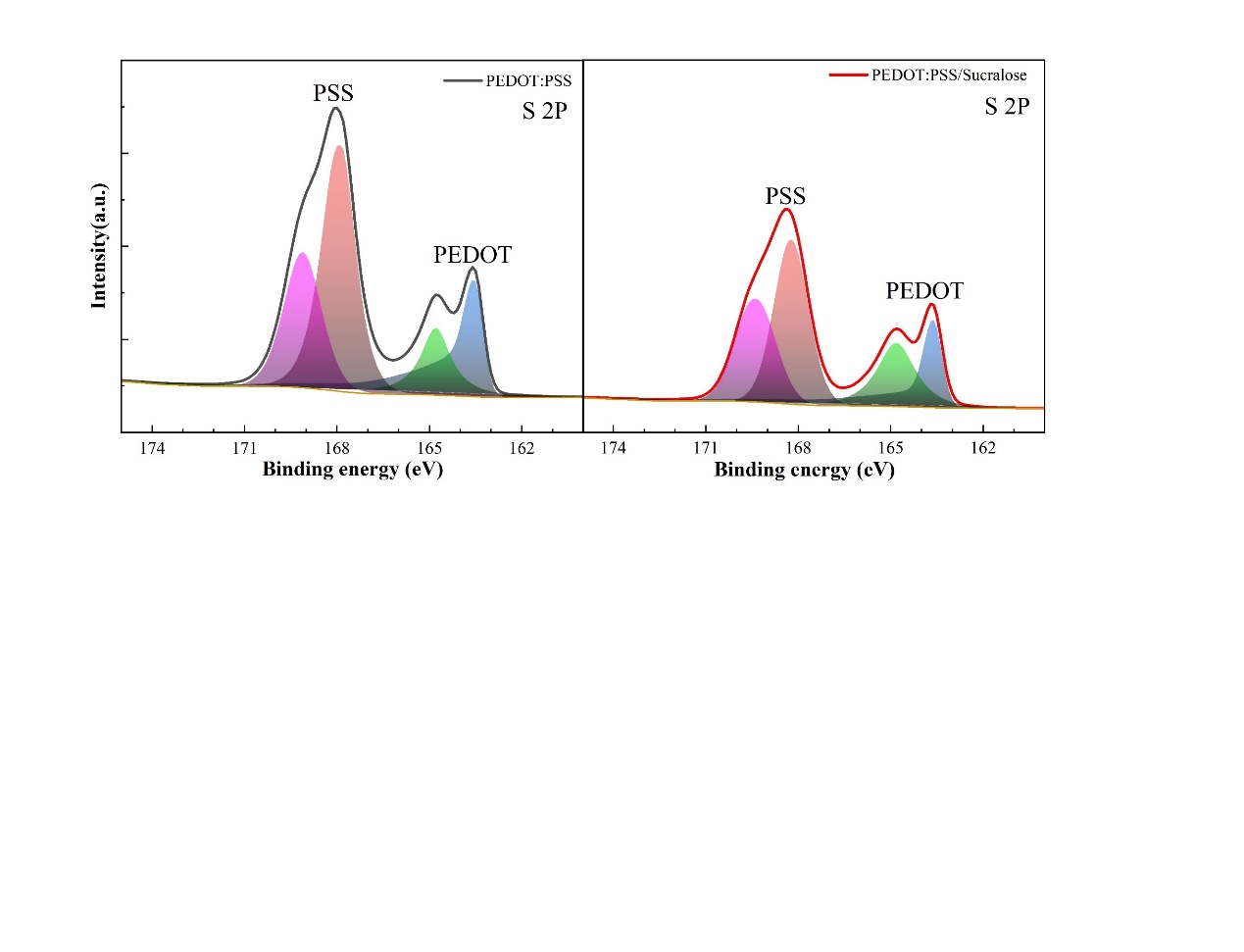


**Figure S13**. S (2p) X-ray photoelectron spectroscopy (XPS) profiles of the surfaces of PEDOT:PSS without and with sucralose.

**Note 3. The XPS analysis of PEDOT:PSS without and with sucralose**

XPS analysis was conducted to confirm the enhanced conductivity of PEDOT:PSS films with addition of sucralose. The peak between 172 eV and 166 eV are assigned to the sulfonate groups in PSS, whereas those within 166-163 eV range can be ascribed to the sulfur atoms in PEDOT (Adv. Funct. Mater. 2021, 31, 2107250). The PSS-PEDOT area ratios for PEDOT:PSS films without and with sucralose were calculated to be 1.858 and 1.682, respectively. The higher PEDOT content in PEDOT:PSS films could result in higher conductivity. The less PSS in films could be due to the loss during the spin-coating process with the help of addition of sucralose leading to phase separation between PEDOT and PSS.


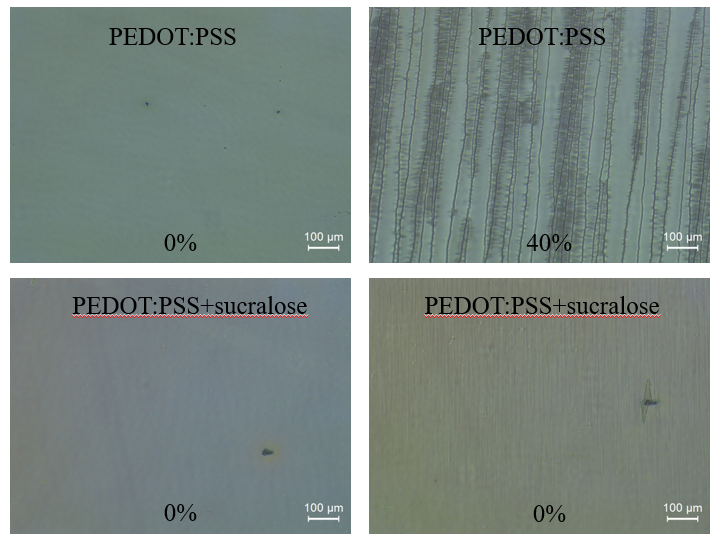


**Figure S14**. Optical microscopy image of PEDOT:PSS film without (up) and with (down) sucralose before and after 40% tensile strain.


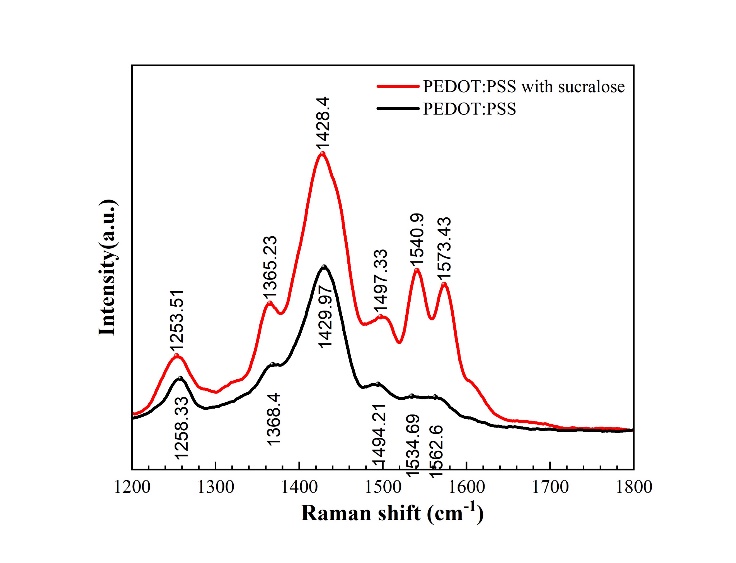


**Figure S15**. Raman spectra of PEDOT:PSS films with and without sucralose.


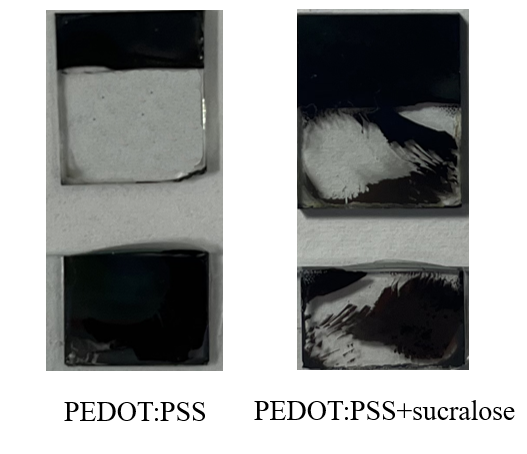


**Figure S16**. The photographs of perovskite-PEDOT:PSS interfaces by peeling off perovskite films from PEDOT:PSS.


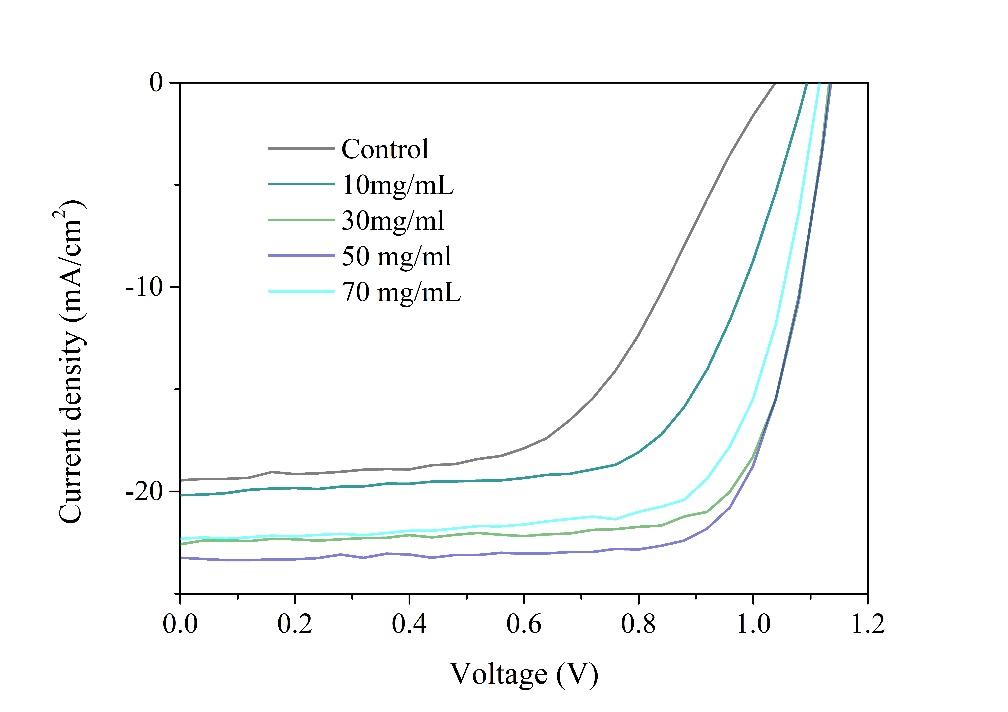


**Figure S17**. J-V curves of f-PSCs based on PEDOT:PSS electrodes with different concentration of sucralose.


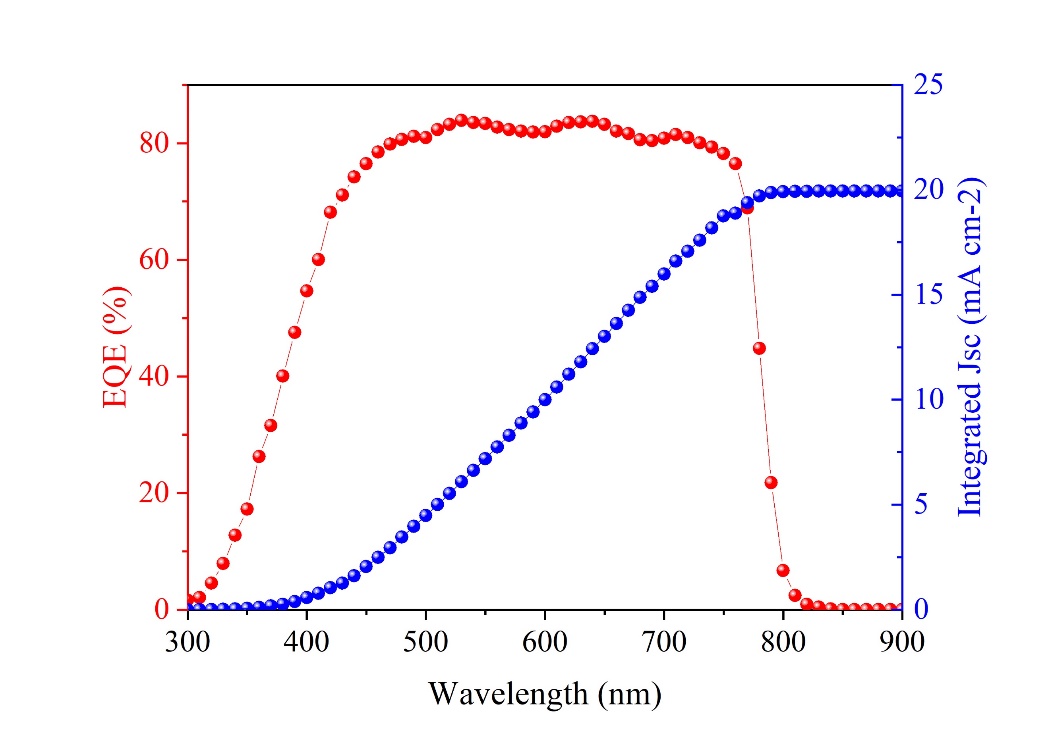


**Figure S18**. The EQE curve of the flexible device with PEDOT:PSS electrode. The integrated short-circuit current density is 20.0 mA/cm^2^.


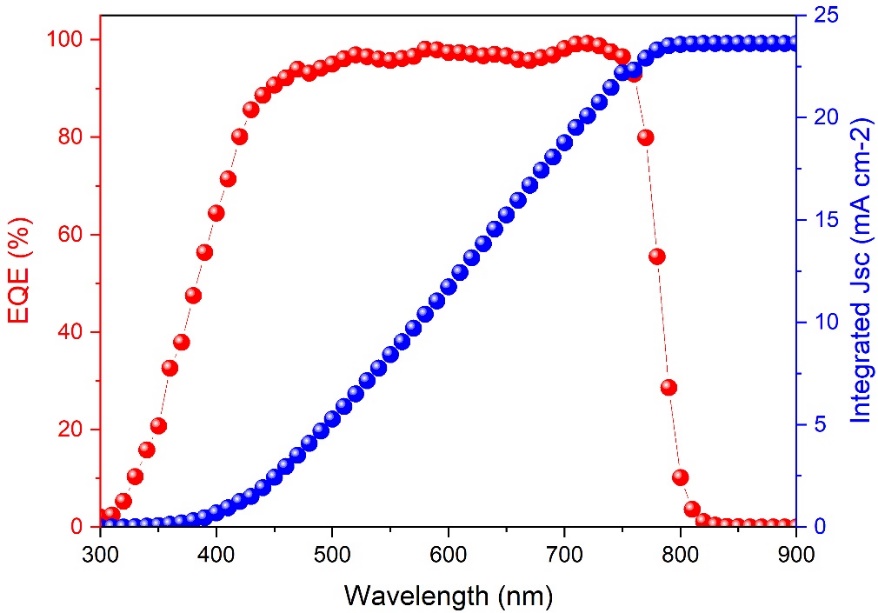


**Figure S19**. The EQE curve of the flexible device with sucralose doped PEDOT:PSS electrode. The integrated short-circuit current density is 23.6 mA/cm^2^.


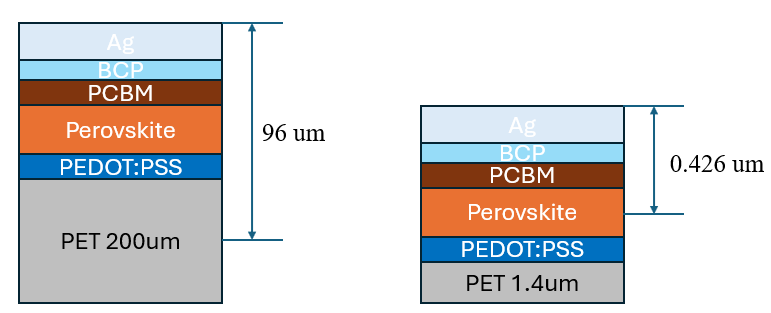


**Figure S20**. The schematic illustration of the distance of the neutral plane from the top surface of flexible perovskite solar cells base on PET substrates with different thickness.

**Note 4. The XPS analysis of PEDOT:PSS without and with sucralose**

The location of neutral planes in f-PSCs can be determined according to the equation (1) (Energy Environ. Sci., 2019, 12, 3182-3191)

$h=\frac{\sum_{i=1}^{n} E_{i}t_{i}[\left( \sum_{j=1}^{i} t_{j} \right)-\frac{t_{i}}{2}]}{\sum_{i=1}^{n} E_{i}t_{i}}$ (1)

where h denotes the distance of the neutral plane from the top surface, *i* is the index of each layer, and E_i_ and t_i_ are the elastic modulus and thickness of each layer *i*, respectively. The elastic modulus and the thickness of each layer are summarized in **Table S5**. As a result, a PET with a thickness of 200 um enables the neutral plane to locate at the middle of PET substrate in f-PSCs (*h*=96 um). In contrast, a PET with thickness of 1.4 um shifts the neutral plane into perovskite layer in f-PSCs (*h*=0.46 um). Therefore, an ultrathin substate could shift the neutral plane into the perovskite layer to significantly improve the mechanical flexibility of flexible perovskite solar cells.


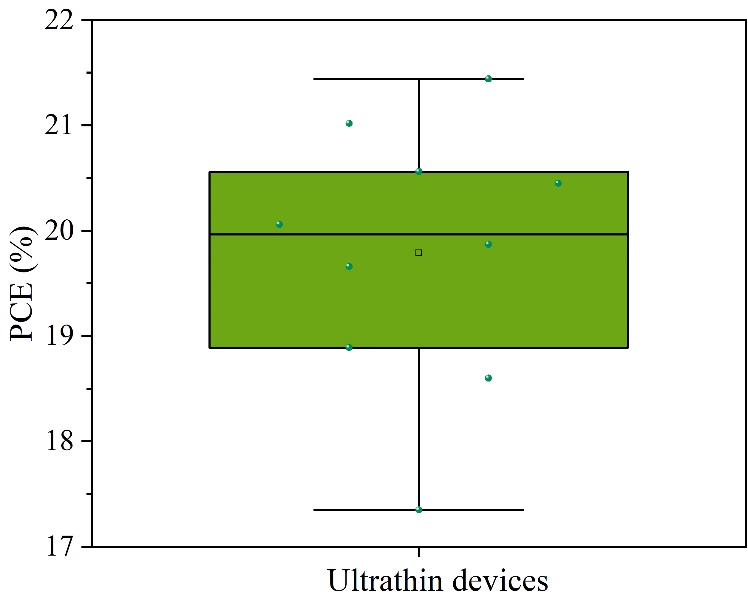


**Figure S21**. The PCE distribution of ten ultrathin devices.


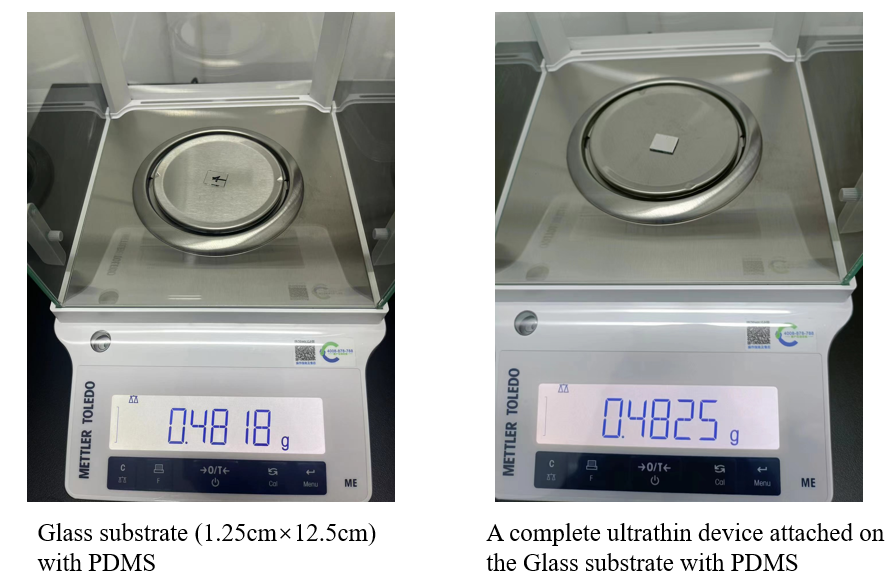


**Figure S22**. The weight of glass substrates with PDMS before and after the deposition of an ultrathin device.


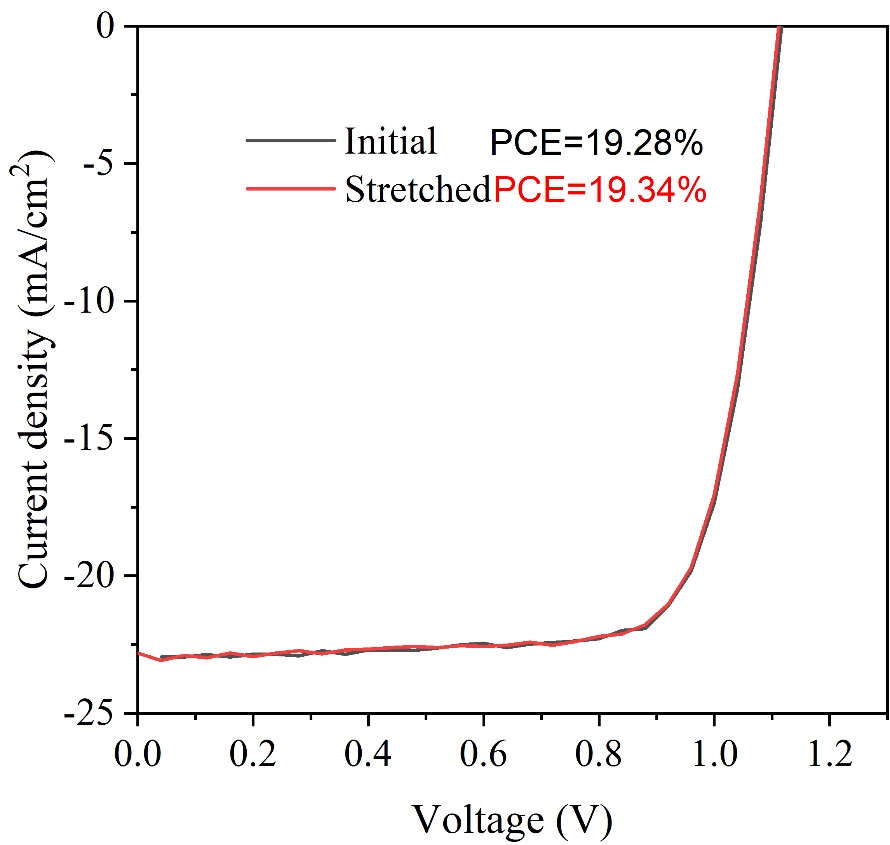


**Figure S23**. The photovoltaic performances of an ultrathin device before and after 1000 times stretching test.


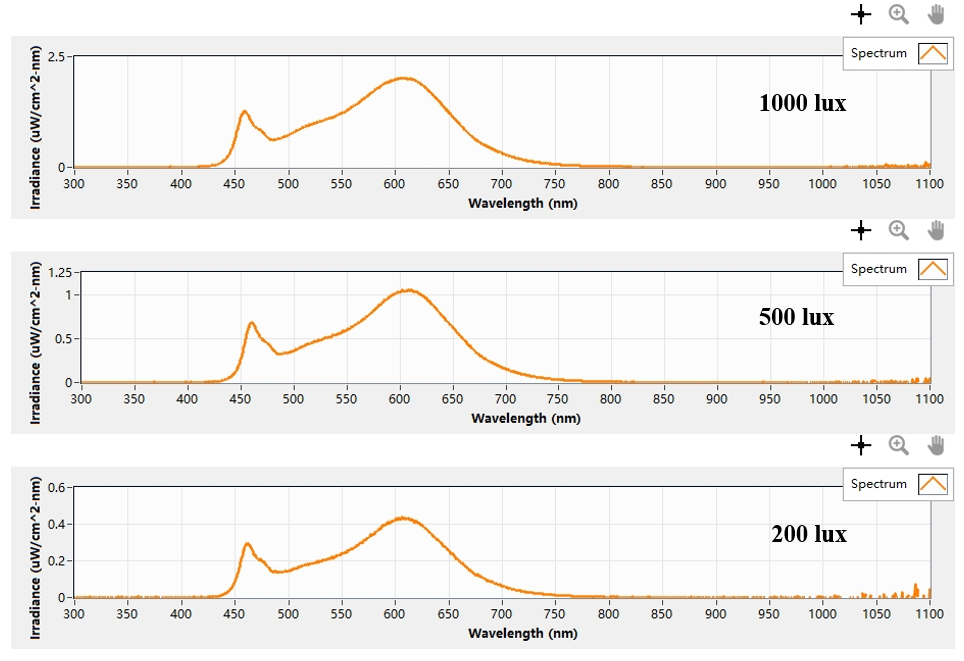


**Figure S24**. The spectrum of LED light with 1000 lux, 500 lux and 200 lux, respectively.

**Table S1**. The detailed parameters of J-V curves of FPSCs with different amounts of PEA_2_PbI_4_.

| Concentration (mol%) | Voc (V) | Jsc (mA/cm^2^) | FF (%) | PCE (%) |
| --- | --- | --- | --- | --- |
| 0 | 1.07 | 19.57 | 73.54 | 15.40 |
| 0.25 | 1.11 | 20.91 | 76.13 | 17.67 |
| 0.5 | 1.12 | 21.16 | 78.31 | 18.56 |
| 1.0 | 1.13 | 20.54 | 77.93 | 18.09 |
| 2.0 | 1.09 | 15.56 | 70.22 | 11.91 |
| 4.0 | 0.96 | 7.60 | 31.80 | 2.32 |

**Table S2**. The detailed parameters of J-V curves of FPSCs based on mixed perovskites with different amounts of PEA_2_PbI_4._

| Concentration (mol%) | Voc (V) | Jsc (mA/cm2) | FF (%) | PCE (%) |
| --- | --- | --- | --- | --- |
| 0 | 1.03 | 24.45 | 77.75 | 19.58 |
| 0.25 | 1.14 | 23.15 | 80.63 | 21.28 |
| 0.5 | 1.16 | 24.20 | 80.58 | 22.90 |
| 1.0 | 1.13 | 21.50 | 80.30 | 19.51 |
| 2.0 | 1.08 | 20.19 | 65.08 | 14.19 |

**Table S3.** The detailed parameters of TRPL of MAPbI_3_ without and with 0.5% of PEA_2_PbI_4_.

| Type of film | τ_1_ (ns) | τ_2_ (ns) | τ (ns) | A1 | A2 |
| --- | --- | --- | --- | --- | --- |
| MAPbI_3_ | 22.89 | 5.31 | 7.6 | 0.37 | 10.63 |
| MAPbI_3_·0.5%PEA_2_PbI_4_ | 22.31 | 149.77 | 134.1 | 0.59 | 0.62 |

**Table S4.** Summary of representative additives for spin-coating PEDOT:PSS film as electrode in photovoltaic devices.

| Additive | Conductivity | Reference |
| --- | --- | --- |
| DMSO | 625 | [1] |
| EG | 1330 | [7] |
| Ionic liquids | 2084 | [8] |
| sucralose | 2500 | This work |

**Table S5.** Elastic modulus and thickness of layers in flexible perovskite solar cells.

|  | Ag | BCP | PCBM | Perovskite | PEDOT:PSS | PET |
| --- | --- | --- | --- | --- | --- | --- |
| Elastic modulus (Gpa) | 76 | 12 | 12 | 18.5 | 3 | 2.5 |
| Thickness (nm) | 150 | 10 | 40 | 500 | 120 | 1400 |

**Table S6**. Photovoltaic performance of typical ultrathin perovskite solar cells

| Device architecture | *V*_OC_  [V] | *J*_SC_  [mA cm^-2^] | FF (%) | PCE [%] | Ref. | Year |
| --- | --- | --- | --- | --- | --- | --- |
| PET/PEDOT:PSS/  CH_3_NH_3_PbI_3_/PTCDI/Cr_2_O_3_/Cr/Au | 0.93 | 17.5 | 76 | 12 | [1] | 2015 |
| PET/ITO/NiOx/CH_3_NH_3_PbI_3_/C_60_/Bis-C_60_/Ag | 0.975 | 20.90 | 69.6 | 14.19 | [2] | 2017 |
| PEN/AgNW/PH1000/Al4083/MAPbBr_x_I_3-x_/PCBM/Al | 1.06 | 18.63 | 77 | 15.18 | [3] | 2019 |
| PET/PEDOT:PSS/CH_3_NH_3_PbI_3_/C_60_/BCP/Cu | 0.96 | 22.45 | 79 | 17.03 | [4] | 2019 |
| Parylene/ITGZO/PTAA/Mixed-cation Perovskite/PC61BM/BCP/Cu | 1.10 | 23.9 | 77 | 20.02 | [5] | 2022 |
| PET/AlOx/PEDOT/Perovkite/PCBM/TiOx/Metal | 1.15 | 22.4 | 78 | 20.1 | [6] | 2024 |
| PET/PEDOT:SS with D-sorbitol/PTAA/PFN/Mix-cation Perovskite/PCBM/BCP/Ag | 1.13 | 23.65 | 79.94 | 21.44 | This work | 2024 |
|  | | | | | | |

**Table S7**. Photovoltaic parameters of device under different light.

| Light | PCE (%) | Jsc (mA/cm^2^) | Voc (V) | FF (%) |
| --- | --- | --- | --- | --- |
| 1000 lux | 36.25 | 0.141 | 0.928 | 83.12 |
| 500 lux | 33.42 | 0.0729 | 0.927 | 78.13 |
| 200 lux | 32.42 | 0.03507 | 0.899 | 63.68 |

**Reference**

[1] M. Kaltenbrunner, G. Adam, E. D. Glowacki, M. Drack, R. Schwodiauer, L. Leonat, D. H. Apaydin, H. Groiss, M. C. Scharber, M. S. White, N. S. Sariciftci, S. Bauer, Nat. Mater. **2015**, *14, 1032-1039.*

[2] H. Zhang, J. Q. Cheng, D. Li, F. Lin, J. Mao, C. J. Liang, K. Y. Jen, M. Gratzel, W. C. H. Choy, *Adv. Mater*. **2017**, *29, 1604695.*

[3] S. Kang, J. Jeong, S. Cho, Y. J. Yoon, S. Y. Park, S. D. Lim, J. Y. Kim, H. Ko, *J. Mater. Chem. A* **2019**, *7, 1107-1114.*

[4] G. H. Lee, M. C. Kim, Y. W. Choi, N. Y. Ahn, J. H. Jang, J. J. Yoon, S. M. Kim, J. G. Lee, D. Kang, H. S. Jung, M. Choi, *Energy Environ. Sci.* **2019**, *12, 3182-3191.*

[5] J. Wu, P. Chen, H. Xu, M. T. Yu, L. Li, H. M. Yan, Y. M. Huangfu, Y. Xiao, X. Y. Yang, L. C. Zhao, W. Wang, Q. H. Gong, R. Zhu, *Sci. China Mater*. **2022**, *65, 2319-2324.*

[6] B. Hailegnaw, S. Demchyshyn, C. Putz, L. E. Lehner, F. Mayr, D. Schiller, R. Pruckner, M. Cobet, D. Ziss, T. M. Krieger, A. Rastelli, N. S. Sariciftci, M. C. Scharber, M. Kaltenbrunner, *Nat. Energy* **2024**, *9, 677-690.*

[7] X. Fan, J. Z. Wang, H. B. Wang, X. Liu, H.; Wang, *ACS Appl. Mater. Interfaces* **2015**, *7, 16287-16295.*

[8] C. Badre, L. Marquant, A. M. Alsayed, L. A. Hough, *Adv. Funct. Mater.* **2012**, *22, 2723-2727.*
